# The Effect of Taichi Practice on Attenuating Bone Mineral Density Loss: A Systematic Review and Meta-Analysis of Randomized Controlled Trials

**DOI:** 10.3390/ijerph14091000

**Published:** 2017-09-01

**Authors:** Liye Zou, Chaoyi Wang, Kevin Chen, Yankai Shu, Xiaoan Chen, Lin Luo, Xitang Zhao

**Affiliations:** 1 Psychosomatic Laboratory, Springfield College, Springfield, MA 01109, USA; lzou@springfieldcollege.edu; 2 College of Sports Science, Jishou 416000, China; yankaishu@yahoo.com (Y.S.); anneychen5748@sina.com (X.C.); 3 College of Physical Education, Jilin University, Changchun 130022, China; chaoyi@jlu.edu.cn; 4 Integrative Medicine Lab, University of Maryland, Baltimore, MD 21201, USA; kchen@som.umaryland.edu; 5 Department of Physical Education, North China Electric Power University, Beijing 102206, China; 6 Department of Physical Education, ZhaoQing University, Zhaoqing 526061, China; xzhao3@springfieldcollege.edu

**Keywords:** Taichi, Taijiquan, bone mineral density, meta-analysis

## Abstract

*Objective*: The purpose of this study was to determine the effects of practicing Taichi on attenuating bone mineral density (BMD) loss. *Methods:* Both electronic and manual searches were performed for randomized controlled trials (RCTs) examining Taichi for bone health. Two review authors independently performed study selection and data extraction according to inclusion criteria. A third party (Lin Luo) emerged to discuss with the two review authors and resolve a disagreement. *Results:* Twenty RCTs were found to meet the inclusion criteria and used for meta-analysis with a total effective sample of 1604. The aggregated results from this systematic review have shown significant benefits in favour of Taichi on BMD at lumbar spine (Standard Mean Difference, SMD) = 0.29; 95% CI 0.15 to 0.43; *p* < 0.0001), femur neck (SMD = 0.56; 95% CI 0.38 to 0.75; *p* < 0.00001), femur trochanter (SMD = 0.04; 95% CI 0.01 to 0.07; *p* = 0.007), total hip BMD (SMD = 0.46; 95% CI 0.16 to 0.76; *p* = 0.003). *Conclusions*: The aggregated results from this systematic review suggests that Taichi is effective on attenuating BMD loss at the regions of lumbar spine and proximal femur neck in special populations (e.g., older adults, perimenopausal and postmenopausal women, people with osteoarthritis, and cancer survivors). Researchers should further examine the effect of Taichi on the proximal femur trochanter and total hip so that a more definitive claim can be made regarding the beneficial effects for attenuating BMD loss in these musculoskeletal regions.

## 1. Introduction

Taichi is a traditional Chinese Qigong exercise that originates from ancient China. It typically consists of a series of distinct movements called Taichi Quan, Taichi sword, Taichi fan, Taichi softball, and Taichi push hand. Such Taichi exercises share the common Chinese medicine theory and place an emphasis on a combination of physical exercise with mental focus; interaction between physical postures and movements, meditative mind, musculoskeletal relaxation and breathing techniques in a harmonious manner [1]. 

Taichi has gained more popularity worldwide since the Chinese Health-Qigong Association was established in 2001 to strive in promoting Qigong [2]. According to the U.S National Health Interview Survey [3], it was estimated that approximately 131 million US citizens have practiced at least one form of mind-body exercises (e.g., Taichi, Qigong, or Yoga) in the past 12 months. As the number of Taichi practitioners has grown in different regions of the globe, more and more researchers have paid attention to this mind-body practice, and utilized scientific methods to investigate the health benefits resulting from practicing Taichi exercises. These studies have examined the effects of Taichi exercises on a variety of health-related parameters across people with different conditions relating to bone mineral density loss, including people with knee osteoarthritis [4,5], and people with fibromyalgia [6,7], people with rheumatic diseases [8,9], people with multiple sclerosis [10], older people with impaired postural stability [11,12], and people with osteopenia or osteoporosis [13]. While existing literature arising from these studies indicates the beneficial effects of Taichi for these special populations, systematic reviews were subsequently conducted for making a definitive claim [14,15,16]. 

Up till now, only three systematic reviews have been published examining the protective effect of Taichi for people who are experiencing bone mineral density (BMD) loss, which are either now outdated or only focused on postmenopausal women [17,18,19]. In the latest systematic review, researchers utilized a meta-analytic method and synthesized the existing evidence, but it is worth noting that sharing a control between two Taichi intervention groups (i.e., double counting participants in the control) resulted in a unit-of-analysis error that was not considered [18]. In addition, because of a small number of randomized controlled trials (RCTs) that existed in the previous systematic reviews, a definitive conclusion relating to Taichi to bone health is difficult to draw. More recently published randomized controlled studies make an updated systematic review necessary, and possibly more conclusive about the effect of Taichi on attenuating BMD loss in older adults [20,21,22,23], people with osteoarthritis [24], hyperlipidemia [25], cancer survivor [26], and perimenopausal women [27]. Given the fact that the substantial number of studies was produced in recent years on the bone health effects of practicing Taichi, it is valuable for the research community to have access to a comprehensive review and summary of these study results. Furthermore, meta-analysis is thought of as the highest level of evidence within the hierarchical structure model and is able to estimate magnitude of the protective effect (resulting from practicing Taichi) for BMD by synthesizing the study findings of RCTs [28], therefore, we have conducted a systematic review and meta-analysis of existing RCTs, to determine whether Taichi training is effective on attenuating BMD loss in people who are experiencing it. 

## 2. Methods

### 2.1. Data Sources and Search Strategy

Electronic literature searches were conducted using Google Scholar, PubMed, Science Citation Index (SCI), Cochrane Library, Scopus, Web of Science, the WHO International Clinical Trials Registry Platform, China National Knowledge Infrastructure (CNKI), and the Wan Fang Database. The following keywords were used individually or in a combined manner for the electronic searches: Taichi (e.g., Taichi Quan, Taichi push hand, Taichi softball, Taiji, Taiji Quan, Taiji push hand, Taiji softball), bone health, bone density, bone mineral density, bone mass, bone strength, bone tissue, bone metabolism, bone biomarkers, and bone turnover markers. After completing the electronic searches, manual searches were subsequently performed through the reference lists of original and review articles. The Preferred Repointing Items for Systematic Reviews and Meta-Analyses (PRISMA) approach was used to present detailed information in this systematic review and meta-analysis [29]. 

### 2.2. Inclusion Criteria

Studies were included if they met the following criteria: (1) RCTs; (2) peer-reviewed studies published in English or Chinese between January 1990 to December 2016; (3) Taichi (e.g., Taichi Quan, Taichi push hand, Taichi softball, or Taichi fan exercise) as a main intervention for bone health; (4) studies including at least one outcome measure relating to BMD (primary outcome) at lumbar spine, proximal femur (trochanter or neck), total hip, total body, or bone biochemical turnover markers (secondary outcome). To determine the effects of Taichi for bone health, the following comparisons in the RCTs are acceptable in this systematic review: (1) Taichi versus no treatment; (2) Taichi + placebo versus placebo; (3) Taichi versus other low-to-moderate walking; (4) Taichi + usual care versus usual care (e.g., exact same amount of calcium supplements were used in both experimental and control groups during intervention period, we assumed the effect of calcium supplements on BMD between the two groups were equivalent. In addition, usage of calcium supplements is reasonable in the studies according to the ethical perspective). Review articles, conference abstracts, magazines, monographs, and videos were excluded. 

### 2.3. Data Selection

The leading review author (Liye Zou) of this present study first created three GoogleDrive electronic folders named “relevant”, “irrelevant” and “unsure.” According to the predetermined inclusion criteria, two review authors (Liye Zou and Chaoyi Wang) independently read through the title and abstract of identified studies and determined which named folder they should belong to. The inter-rater reliability about the eligible studies within “the relevant folder” was calculated according to the agreement percentage in two-rater model [30]. A third party (Lin Luo) discussed with the two reviewer authors and resolved any disagreement about the placement of articles in the “unsure folder.” 

### 2.4. Data Extraction

Two review authors (Liye Zou and Chaoyi Wang) independently extracted detailed data from each eligible study according to a pre-designed summary table evaluating the effect of Taichi intervention on BMD. Table 1 includes name of author and year of publication, study design, study location, study participants, intervention and sample size, outcomes measured, and results. A third party (Lin Luo) ensured the consistency of the data extracted by the two review authors. 

For meta-analysis, with regard to the within-group change scores (mean and standard deviation) for the interesting outcome measures, if studies did not report the change score data, the leading review author (Liye Zou) first tried to reach out to the corresponding author and asked for the original dataset. In cases where the data was not obtainable, the leading review author utilized one of the following methods: (1) if no significant difference on the interesting outcome measure at baseline between two groups was observed, post-intervention score was utilized for data analysis; (2) if a significant difference on the interesting outcome at baseline between two groups was observed, the leading review author (Liye Zou) tried to estimate the change score and standard deviation through standard formulas provided by Cochrane Handbook for Systematic Reviews of Interventions [31,32]. If review authors were unable to find the relevant information for estimating the change scores and standard deviations, the study was excluded. A special case is that if a RCT contained two Taichi-based interventions (Taichi Quan and Taichi push hand, Taichi Quan and Taichi softball, or Taichi softball and Taichi push hand) and a control group. According to Higgins and Green [33], it is recommended to combine groups (two Taichi-based intervention groups). If two comparison groups (non-Taichi and control groups) were included in the eligible studies, reviewers kept the control group but removed the non-Taichi group. 

### 2.5. Methodological Quality of Assessment 

Strictly speaking, every RCT should be double-blinded. However, Taichi as a mind-body exercise to be used in RCTs it is reasonable not to be blinded to participants and Taichi instructors. Therefore, a modified Jadad Scale was utilized, including random allocation, random assignment, eligibility criteria, outcome assessors blinded, withdrawal and dropouts reported, sample size justified/estimated, appropriate data analysis, Taichi intervention described, and Qualifications of Taichi instructors [33]. If a criterion was met, a point (one) is awarded for the study and vice versa (zero). For each study included, a sum score ranging from one to nine could be obtained, with higher scores indicating better methodological quality. The sum score is classified methodological quality of each study into: (1) poor quality = score ≤ 3; (2) fair quality = score between 4 and 6; (3) good quality = score between 7 and 9 [34]. 

### 2.6. Statistical Analysis

In this systematic review, BMD as primary outcome measures was used for meta-analysis. For bone turnover markers, if homogeneity of outcome measures across the eligible studies existed, we also have conducted a meta-analysis for each individual biomarker. If not, we only descriptively reported the results of the bone turnover markers. Revman 5.3 software within the Cochrane Collaboration for data analysis were employed to synthesize the mean change scores in BMD and bone turnover markers. Review authors selected the standardized difference approach to measure the effect size (ES). In the meanwhile, a fixed-effect model in this meta-analysis was performed along with 95% confidence intervals (CI) and weighted mean differences. The chi-square test and the Higgins *I*^2^ test was used to evaluate heterogeneity of the outcome measures across the eligible studies. 

## 3. Results

### 3.1. Literature Search

**Figure 1:**
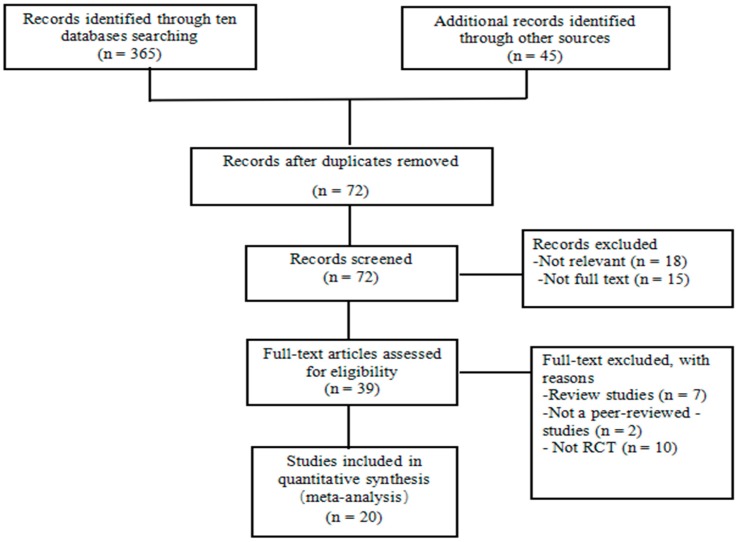
Flowchart showing the retrieval of studies for review.

A total of 410 potential relevant records were identified through both the electronic and manual searches. According to the title and author name, 72 articles remained after removing the 338 duplicates and unqualified articles. Thirty three articles were excluded because they were not relevant (n = 18) or not full text (n = 15). Nineteen full-text articles were excluded because they were review studies (n = 7), not peer-reviewed studies (n = 2), and not RCTs (n = 10). The final number of 20 randomized controlled trials (RCT) was used for meta-analysis. Of these, eleven studies were published in English [21,22,23,26,35,36,37,38,39,40,41] and nine in Chinese [20,24,25,27,42,43,44,45,46]. In terms of the searching terms, there are two studies relating to Taichi push hand [44,46], one study relating to Taichi soft-ball [27], and seventeen studies relating to Taichi Quan [20,21,22,23,24,25,26,35,36,37,38,39,40,41,42,43,45]. The flowchart showing the retrieval of studies for this review is displayed in Figure 1.

### 3.2. Study Characteristics

The characteristics of the eligible 20 RCTs are presented in Table 1. These studies were published between 2004 and 2015. A total of 1604 participants (an age range from 45 to 79 years old) was included in this systematic review, with a sample size of an individual study ranging from 16 to 253 participants. Special populations were recruited in the eligible studies investigating Taichi for bone health, including middle-aged and older adults, perimenopausal and postmenopausal women, breast cancer survivors, people with osteoporosis, and women with osteoarthritis. When compared to the Taichi exercise intervention group, the condition in control group varies by study, including placebo [37,38], standard care/calcium supplement/Green tea polyphenols supplementation [26,37,38,40,42,43,46], original and sedentary lifestyle [20,23,25,27,35,36,39,41,42,44,45], self-help education [24], resistance training [21], recreational activities (e.g., walking or dancing) [22]. Study participants in the Taichi exercise intervention groups (intervention duration from 12 weeks to 12 months) experienced different types of Taichi (e.g., Yang-style, Chen style, Taichi push hand, Taichi softball, and Taichi). The Taichi training duration ranged from 45 to 90 min, along with the frequency of weekly sessions from 2 to 7. Adverse events were reported in some, but not all studies.

### 3.3. Methodological Quality 

The inter-rater reliability about the eligible studies within “the relevant folder” was 95%. According to the modified Jadad Scale, a methodological quality of the eligible randomized controlled studies ranged from 3 to 9 points, with a higher score indicating better methodological quality (Table 2). Only two studies scored 9 points [36,40]. Points in most of the eligible studies were deducted because of absence of randomization methods (e.g., computerized generator) in the 12 RCTs [20,22,23,25,27,35,39,42,43,44,45,46], eligible criteria [23], outcome assessor blinded [20,22,23,24,25,26,27,35,37,39,41,42,43,44,45,46], sample size estimated [20,21,22,23,25,26,27,37,38,39,42,43,44,45,46], appropriate data analysis [20,22,23,25,27,42,43,44,45,46], Taichi intervention described [41,43], and qualification of Taichi instructor [22,23,35,41,44,45,46]. 

### 3.4. Meta-Analysis of Outcomes Measured 

For meta-analysis, two pair-wise comparisons are present in the same eligible study. If a study included four groups [Taichi group, Taichi + standard care/supplement, standard care, control group], two pair-wise comparisons are as follow: Taichi vs. control groups and Taichi + standard care/supplement vs. standard care. 

**Figure 2:**
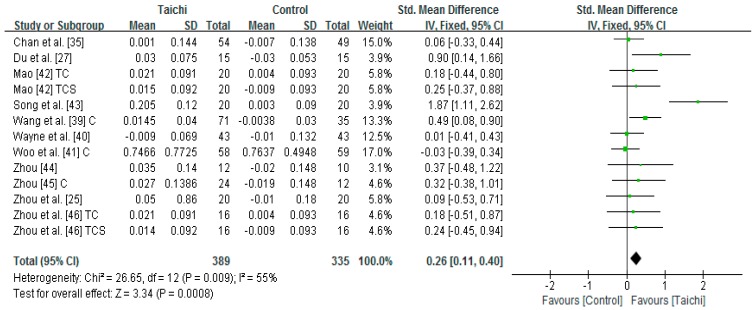
The effect of Taichi on lumbar spine (TC = Taichi vs. original lifestyle; TCS = Taichi + supplement vs. supplement; C = combined two Taichi-based interventions).

Eleven studies (13 pair-wise comparisons) examined the effect of Taichi on lumbar spine measured by Dual-energy X-ray densitometer or Dual-energy X-ray Absorptiometry [25,27,35,39,40,42,43,44,45,46]. A higher positive value of the mean change score for the lumbar spine indicates an increase of BMD, whereas a higher negative value of the mean change score for the lumbar spine indicates the BMD loss. The aggregated result has shown a significant benefit in favour of Taichi on the lumbar spine BMD (SMD = 0.26; 95% CI 0.11 to 0.40; *p* = 0.0008) (Figure 2). 

**Figure 3:**
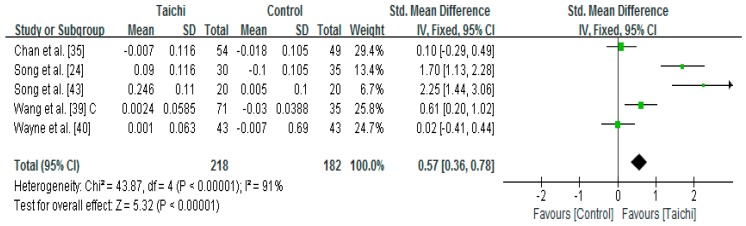
The effect of Taichi on proximal femur neck (C = combined two Taichi-based interventions).

Five studies examined the effect of Taichi on proximal femur neck measured by Dual-energy X-ray densitometer or Dual-energy X-ray Absorptiometry [24,35,39,40,43]. A higher positive value of the mean change score for the proximal femur neck indicates an increase of BMD, whereas a higher negative value of the mean change score for the femur neck indicates the BMD loss. The aggregated result has shown a significant benefit in favour of Taichi on the proximal femur neck BMD (SMD = 0.57; 95% CI 0.36 to 0.78; *p* < 0.00001) (Figure 3).

**Figure 4:**
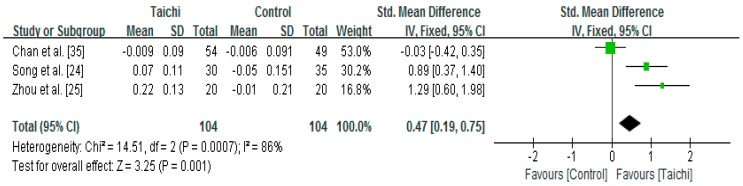
The effect of Taichi on proximal femur trochanter.

Three studies examined the effect of Taichi on proximal femur trochanter measured by Dual-energy X-ray densitometer or Dual-energy X-ray Absorptiometry [24,25,35]. A higher positive value of the mean change score for the femur trochanter indicates an increase of BMD, whereas a higher negative value of the mean change score for the femur trochanter indicates the BMD loss. The aggregated result has shown a significant benefit in favour of Taichi on the proximal femur trochanter BMD (SMD = 0.47; 95% CI 0.19 to 0.75; *p* = 0.001) (Figure 4). 

Researchers compared Taichi with control group (original lifestyle) on total body bone mineral content (BMC) measured by Dual-energy X-ray densitometer or Dual-energy X-ray Absorptiometry [27,36]. A higher positive value of the mean change score for the total body indicates an increase of BMD, whereas a higher negative value of the mean change score for the total body indicates the BMD loss. The aggregated result has shown that there was no significant difference between Taichi and control group (original lifestyle) in enhancing the total body BMC (SMD = 0.07; 95% CI −0.16 to 0.30; *I*^2^ = 68%; *p* = 0.56). 

Four studies (five pair-wise comparisons) examined the effect of Taichi on alkaline phosphatase (ALP) measured by enzyme-linked immunosorbent assay (ELISA) [20,27,37,43]. A higher negative value of the mean change score for the ALP indicates the bone formation activity osteoblasts, whereas a higher positive value of the mean change score for the ALP indicates the bone destruction by the uncontrolled activity of osteoclasts. The aggregated result has shown that there was no significant difference between Taichi exercises and no treatment and other standard care (e.g. calcium supplements or green tea polyphenols) in decreasing the serum level of the ALP (SMD = −0.05; 95% CI; −0.30 to 0.19; *I*^2^ = 0%; *p* = 0.67). 

Three studies examined the effect of Taichi on bone-specific alkaline phosphatase (BAP) as a biomarker of bone formation [21,26,38]. A higher positive value of the mean change score for the BAP indicates an increase in the bone-forming activity of osteoblasts, whereas a higher negative value of the mean change score for the BAP indicates the bone destruction by the uncontrolled activity of osteoclasts. The aggregated result has shown a significant benefit in favour of Taichi on the BAP (SMD = 0.59; 95% CI; 0.28 to 0.9; *I*^2^ = 0%; *p* = 0.0002). 

**Figure 5:**
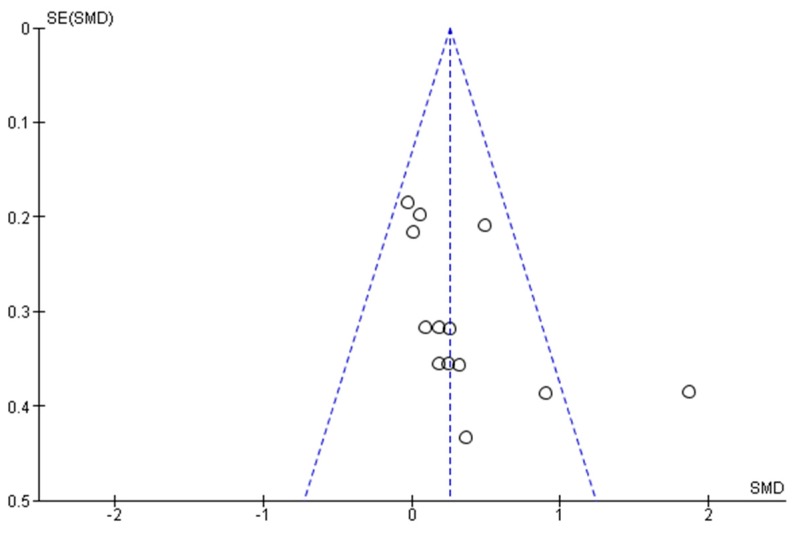
Evaluation of publication bias for lumbar spine.

**Figure 6:**
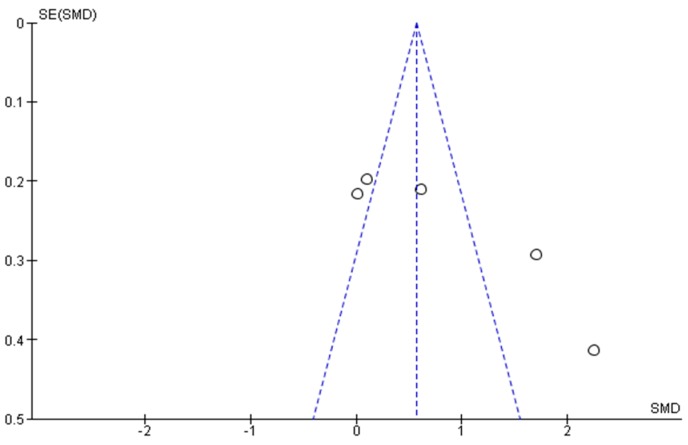
Evaluation of publication for femur neck.

Funnel plots (Figure 5 and Figure 6) suggest that the meta-analyses were likely to be affected by publication bias. 

## 4. Discussion

### 4.1. Summary of Evidence 

The present meta-analysis has suggested that Taichi exercise may be effective in attenuating BMD loss (e.g., lumbar spine, proximal femur neck and trochanter, and total hip) and improving bone biomarkers (BAP and CTX: C-terminal telopeptide of type I collagen) in special populations, including middle-aged and older adults, perimenopausal and postmenopausal women, people with osteoarthritis, breast cancer survivors, and people with osteoporosis. 

### 4.2. Bone Mineral Density 

The eligible studies examining the effects of Taichi on BMD have focused on the specific skeletal regions, including lumbar spine, proximal femur (neck and trochanter), total body, total hip, distal tibia, ultra-distal radius and ulna of wrist, and calcaneus. The aggregated results of the present meta-analysis showed that Taichi is beneficial for lumbar spine, proximal femur (neck and trochanter), and total hip. The magnitude of the effects was statistically significant, indicating the Taichi is an exercise modality that may be utilized as a strategy for attenuating BMD loss. A possible explanation regarding the effects of Taichi on attenuating BMD loss at the specific weight bearing skeletal regions is attributed to the features of Taichi exercise: (1) the waist must be constantly employed as a primary driving force in order to initiate a voluntary, correct Taichi movement, which facilitates shear forces at the lumbar spine; (2) neutralizing incoming forces is needed for maintaining the entire body in equilibrium while performing a constant shift of weight from one single leg to another, which produces the foot-floor impact force [47,48]. Both the shear force and the foot-floor impact force may possibly become mechanical loading stimulation for the specific weight-bearing skeletons (e.g., lumbar spine, proximal femur neck and trochanter, and total hip), rather than total body BMD (the reason why the positive effects of Taichi on retarding total body BMD loss was not observed from the aggregated results of the present meta-analysis). 

The aggregated result has shown a significant benefit in favour of Taichi exercise for attenuating the total hip BMD. It is worth noting that from gender perspective, only female Taichi group is shown to have positive effect on attenuating total hip BMD, as compared to female counterparts in control group [41]. It may be attributed to that the intensity of Taichi as a low-to-moderate impact exercise for men is insufficient, as compared to women who have a relative lower threshold of exercise intensity. In addition, although the eligible studies individually reported the beneficial effects of Taichi for retarding the distal radius of wrist BMD [25,45] and improving the bone quality index at the calcaneus [20,25], the aggregated results have shown that there were no significant differences between Taichi exercises and other conditions (e.g., no intervention, instructor-led walking) in attenuating these skeletal regions. This may be attributed to a relatively small sample size (ranging from 24 to 64 in the pair-comparison) and inappropriate data analysis. Finally, because only one study has shown the positive effect of Taichi exercise on retarding the BMD loss at ultra-distal tibia (trabecular and cortical compartments) [35], trunk [27], and calcaneus (broadband ultrasound attenuation and speed of sound) [20], the definitive claim with respect to Taichi exercise for retarding these skeletons losses should not be made in this systematic review. 

### 4.3. Bone Turnover Markers

According to National Institutes of Health [49], relationships between BMD and bone biomarkers have been extensively investigated and bone turnover markers can be thought of as predictors to determine whether the osteogenic response takes place. More specifically, there are two types of biomarkers: (1) formation biomarkers (Gla-protein, GP; alkaline phosphase, ALP; bone-specific alkaline phosphatase, BAP; osteocalcein, OSC; procollagen type 1 carboxy-terminal propeptide, and procollagen type 1 amino-terminal propeptide) express the metabolic activity of osteoblasts; (2) resorption biomarkers (serum and urinary pyridinoline, tartrate-resistant acid phosphatase, C-terminal telopeptide of type I collagen, CTX) express the metabolic activity of osteoclasts [50]. Because the heterogeneity of bone biomarkers across a small number of eligible studies existed, the present meta-analysis only synthesized the following biomarkers, including ALP, BAP, OSC, and CTX. With respect to the bone formation biomarkers, a significant improvement on the BAP in the present meta-analysis was identified in favor of Taichi exercise, but ALP and OSC. It is reasonable that a significant improvement was observed on the BAP rather than ALP because the detection of BAP as a specific bone formation marker is more sensitive, whereas the ALP needs to be used with other tests in order to making an accurate detection of bone disorders [51]. With respect to the resorption biomarkers, two studies supported the beneficial effects of Taichi on CTX. Although the positive effects of Taichi on some of the bone turnover markers have been shown in the present meta-analysis, the clinical application is still immature because of the effects of biological variation [50,52]. 

### 4.4. Exercise Intervention 

Although 12-week Taichi intervention has been commonly utilized in research community and shown to improve a variety of health-related parameters (e.g., balance, flexibility, muscular strength, respiratory function, and blood pressure) in different populations (e.g., older adults, hypertension, Parkinson’s disease, and diabetes) [53,54,55,56,57], its protective effect for BMD loss is difficult to be found after a 12-week Taichi intervention because the bone remodeling cycle typically takes at least 24 weeks [50,58]. The majority (73%) of the eligible studies examining the effects of Taichi on BMD in the present meta-analysis followed the 24-week intervention principle, it may be the reason why the encouraging findings were observed. Study participants experienced Taichi training duration ranged from 45 to 90 min, along with the frequency of weekly sessions from 2 to 7. Such training regimens in the eligible studies are largely based on tradition and intuition, a definitive claim regarding specific training frequency of Taichi for attenuating BMD loss is difficult to be made. Therefore, future studies should be conducted to examine the effects of Taichi training frequency/dosage on BMD and establish evidence-based guidelines relating to the Taichi training frequency for maximizing its protective effect for attenuating BMD loss as well as for other special populations. 

Although several different styles of Taichi share the common traditional Chinese medicine theory focusing on a mind-body combination, each style has its unique feature: (1) Yang-style Taichi is characterized by slow, gentle, and extensive stretching movement; (2) Sun-style Taichi is characterized by quick and compact movements; (3) Chen-style Taichi has an emphasis on more strength, quick, and skipping movements [59]. Yang-style is the most commonly used Taichi form as an exercise intervention program in the research community, to determine its effects for health-related parameters. It is worth noting that in the present systematic review, some researchers attempted to use other styles of Taichi form (e.g., Chen-style and Sun-style) and both Sun- and Chen-style Taichi forms have been shown to have the protective effects for attenuating BMD loss. From mechanical loading perspective, Chen-style is more likely to meet the criteria of high-impact, weight-bearing exercise for stimulating bone modeling because it emphasizes powerful, quick, and skipping movements, as compared to Sun-style (modest impact) and Yang-style (low impact). The encouraging findings with respect to the effects of Sun-style and Chen-style Taichi on attenuating BMD loss is thereby reasonable. In addition, Taichi push hand and Taichi softball as advanced styles of Taichi branches have rarely been employed in the research community. In particular, Taichi push hand is similar and comparable to resistance exercise (high-impact, weight-bearing), which may be more effective in stimulating osteogenic response. Wang et al. [39,60] reported that simplified Taichi form (Taichi push hand) is superior to traditional Yang-style for attenuating BMD loss at femoral neck, and lumbar spine. More specifically, when 80% of the eligible studies demonstrated the effects of a long-term Yang-style for retarding BMD loss in comparison to those in control group who demonstrated more BMD loss, Wang et al. found a slight increase of BMD at the three skeletal regions after a long-term Taichi push hand movements. Such findings indicate that Taichi push hand movements may be more suitable for special population who are experiencing BMD loss. 

Therefore, researchers should further confirm the protective effects of these styles (e.g., Sun-style, Chen-style, Taichi push hand, and Taichi softball) for bone health. Compliance of Taichi program and qualification of Taichi instructor may have a direct relationship with the magnitude of protective effects on BMD, which should be further examined as well. For instance, Wayne et al. [40] reported per protocol group (compliance ≥ 75% Taichi intervention session requirement) demonstrated a significant benefit in favor of Taichi in attenuating BMD loss, rather than Taichi group itself. The publication bias reported in this review may be attributed to the following reasons, including the exaggeration of Taichi intervention effects in small studies, location biases (mainland China, Poland, Hongkong, USA, and South Korea), true heterogeneity size of effects differs according to study sample size and intensity of Taichi intervention), and inadequate analysis. 

### 4.5. Study Limitations

Although beneficial effects of Taichi training on attenuating the BMD loss are found in this systematic review, several study limitations should be acknowledged, including the fact of small sample size and variety of design and intervention, quality of previous studies selected (12 of twenty studies were qualified as “fair”), and especially variation in the control group, which made some outcomes less conclusive. From ethical and pragmatic perspectives, calcium supplements/standard care was implemented among people who are experiencing BMD loss, which are reasonable. Ideally, to determine whether Taichi training was effective in attenuating BMD loss, the present meta-analysis should only include pair-comparison: Taichi exercise vs. no treatment. It is noteworthy that calcium supplements/standard care may probably contaminate the effect of Taichi on BMD and bone biomarkers. In addition, given that a control group (original lifestyle or no treatment) was absent in some eligible studies, the present meta-analysis also included pair-comparisons: Taichi vs. walking, this type of pair-comparison may attenuate the beneficial effects of Taichi on outcome measures so that the significant benefit in favor of Taichi exercise is difficult to be detected, particularly for bone biomarkers. 

## 5. Conclusions

The study findings of the present meta-analysis suggest that a long-term (at least 24 week) Taichi training may be an effective intervention to attenuate BMD loss (lumbar spine, proximal femur neck and trochanter) in special population (e.g., perimenopausal and postmenopausal women, older adults, breast cancer survivor, women with osteoarthritis). More high quality randomized controlled studies should be conducted to substantiate the effect of Taichi on retarding BMD loss at the ultra-distal tibia, trunk, calcaneus, and bone biomarkers. In addition to investigating training frequency/dosage of Taichi for maximizing health benefits, particularly the protective effects for BMD, researchers should also investigate the effects of other styles of Taichi (e.g., Chen-style Taichi, Sun-style Taichi, and Taichi push hand) on health-related parameters.

## Figures and Tables

**Table 1:** Summary of the characteristics of the TC studies for bone health and turnover markers.

Author [Reference]	Study Design	Study Location (Language)	Study Participants	Sample Size (Participants/Analyzed) and Intervention	Outcomes Measured	Study Results
Chen et al. [20]	RCT	Shanghai, China (Chinese)	40 female and older adults, aged 55 to 65	TC (20/20): 55 to 65 min, 3 to 4 times weekly for 20 weeks (Yang-style) CG: sedentary lifestyle	Calcaneus ultrasound BMD (BQI, BUA, and SOS) Biomarkers (ALP)	Mean change (Sig) for TC vs. CG: BQI (−3.53 vs. −8.32), SOS (−14.11 vs. −15.57), and BUA (−1.58 vs. −9.21). Mean change (Sig) for TC vs. CG:ALP (−13 vs. −12.69)
Chan et al. [35]	RCT	Hongkong, China (English)	132 postmenopausal women (54.0 ± 3.5 years)	TC (67/54): five 50-min sessions weekly for 12 months (Yang style) CG (65/54): sedentary lifestyle	BMD (Lumbar spine – L2-4, proximal femur [neck and trochanter], and ultral distal tibia [tBMD, iBMD, CTD])	TC vs. CG (*NS*): lumbar spine (0.10 vs. −0.89%), proximal femur neck (−0.94 vs. −1.80%), proximal femur trochanter (−1.19 vs. −0.56%), tBMD (−0.53 vs. −1.46), iBMD (−0.61 vs. −1.58), and CTD (−0.39 vs. −1.40).
Du et al. [27]	RCT	Shanxi, China (Chinese)	30 perimenopausal women, aged 45 to 55	TCSB (15/15): 90 min, 4 to 5 times weekly for 24 weeks. CG: original lifestyle	BMD (Total body, lumbar spine [L1-4], and trunk) Biomarker (ALP)	Mean BMD change (Sig) for TC vs. CG: lumbar spine (0.03 vs. −0.003), total body (0.01 vs. −0.02), and trunk (0 vs. −0.05) Mean change for TC vs. CG: ALP (−1.1 vs. 0.2)
Hui et al. [36]	RCT	Hongkong, China (English)	253 middle-aged participants (45.8 ± 5.3 years)	TC (129/129): five 45-min sessions weekly for 12 weeks (Yang-style) CT (124/124): original lifestyle	BMC (total body)	Mean BMD change (NS) for TC and CG: total body (−0.39 vs. −0.33)
Mao [42]	RCT	Shangxi, China (Chinese)	80 postmenopausal women (56.78 ± 2.91 years)	TC (20/20) and TCS (20/20): 45 to 50 min, 7 times weekly for 20 weeks. CS (20/20): standard care (calcium supplement) CG (20/20): original lifestyle	BMD (Lumbar spine [L2-4])	TC vs. CG and TCCS vs. CS (Sig): Lumbar spine (1.361 vs. −0.874) and (2.036 vs. 0.378), respectively.
Peppone et al. [26]	RCT	USA (English)	16 breast cancer survivors, median age of 53 years	TC (7/7): three 60-min sessions weekly for 12 weeks (Yang-style) CG (9/9): standard care	Biomarkers (NTx, BAP, and BRI)	BAP (NS): TC (8.3 to 10.2; 22.4%) vs. ST (7.6 to 8.1; 6.3%). NTx (Sig): TC (17.6 to 11.1; −36.6%) vs. ST (20.8 to 18.8; –9.6%) BRI (Sig, *p* = 0.05): TC (1.6) vs. ST (0.23)
Shen et al. [21]	RCT	Texas, USA (English)	28 sedentary, older adults. TC (78.8 ± 1.3) RT (79.4 ± 2.2)	TC (14/14) three 40-min sessions weekly for 24 weeks (Yang-style) RT (14/14): three 40-min sessions weekly for 24 weeks (bench press, leg press, leg curl, leg extension, and seated row on a resistance exercise machine, as well as shoulder press and arm curl)	Biomarkers (BAP, PYD, PTH, and BAP/PYD ratio), but mean score and standard deviation were not reported.	After 6 weeks, both TC and RT exhibited higher level of serum BAP, as compared to the baseline and the TC group exhibited a greater increase in serum BAP than the RT group. BAP/PYD ratio was higher than baseline only in the TC group, and the increase of the ratio in the TC was greater than that in the RT group.
Shen et al [37]	RCT	Texas, USA (English)	171 postmenopausal women TC + placebo (58.3 ± 7.7); TC + GTP (57.6 ± 6.7); GTP (56.5 ± 5.5); Placebo (57.6 ± 7.5)	Placebo + TC (42/37): medicinal starch 500 mg daily and 24-move simplified Yang-style TC training (three 60-min sessions weekly for 24 weeks. TC + GPT (38/37): same as TC group + GTP 500 mg daily. GPT (47/39): GTP 500 mg daily Placebo (44/37): same as medicinal starch 500 mg daily.	Biomarker (ALP).	No significant change in the ALP was observed
Shen et al. [38]	RCT	Texas, USA (English)	Same as Shen et al. [37]	Same as Shen et al. [37] Yang-style	Biomarkers (BAP and TRAP)	A significant main effect of TC on serum BAP at 3 months (*p* = 0.04). No significant main effect of TC on TRAP was found.
Song [43]	RCT	Jiangsu, China (Chinese)	40 people with osteoporosis. TC (62.67 ± 11.23) CG (63.81 ± 13.07)	TC (20/20): six 60-min sessions weekly (yang-style, but not report the length of intervention) + standard care CG (20/20): standard care	BMD (lumbar spine [L2-4] and femoral neck) Biomarkers (BGP and ALP)	Mean change (Sig) for TC and CG: lumbar spine (0.205 vs. 0.003) and femoral neck (0.228 vs. 0.005). Mean change (Sig) for TC and CG: and BGP (−2.04 vs. −0.61) and ALP (−17.31 vs. −11.58)
Song et al. [24]	RCT	South Korea (English)	82 women with osteoarthritis TC (mean age = 63 years) CG (mean age = 61 years)	TC (41/30): 60 to 65 min, 7 times weekly for six months (Sun style) CG (41/35): 60-min self-help education session, once monthly for six months	BMD (DXA): Femoral neck and trochanter.	Mean change (Sig) for TC vs. CG: Femur neck (0.09 vs. −0.10), (0.04 vs. −0.04), and trochanter (0.07 vs. −0.05).
Song et al. [22]	RCT	Henan, China (English)	105 community living elderly women, aged 55 to 65.	TC (35/31): Chen Style CG1 (35/33): Dance CG2 (35/30): Walking six 40-min sessions weekly for 12 months in three selected groups	BMD (BQI)	Mean Change (Sig) for TC vs. CG1 vs. CG2: BQI (10.51 vs. 7.65 vs. 7.69)
Sufinowicz et al. [23]	RCT	Poland (English)	90 men aged over 60 (68.83 ± 5.84 years)	TC (35/35): 45-min, twice per week for four months CG (55/55): original lifestyle	Biomarkers (OSC and CTX)	Mean change (Sig) for TC vs. CG: CTX (−0.31 vs. −0.065) and OSC (−0.949 vs. −0.751)
Wang et al. [39]	RCT	Shanghai, China (English)	119 postmenopausal women, aged 52 to 65	TC (40/34): four 60-min sessions weekly for 12 months TCRT (40/37): four 60-min sessions weekly for 12 months CG (39/35): original lifestyle	BMD (lumbar spine [L2-4] and femoral neck)	Mean change (Sig) for TCRT vs. TC vs. CG: Lumbar spine (0.0182 vs. 0.0105 vs. −0.0038), femur neck (0.0004 vs. 0.0045 vs. −0.03), and (−0.0047 vs. −0.0171 vs. −0.0397)
Wayne et al. [40]	RCT	Boston, MA, USA (English)	86 post-menopausal osteopenic women, aged 45 to 70	TC (43/42): 99.5 h during 9-month intervention plus standard care. Of the TC group, 26 completed 75% training requirements or above as TCAG CG (43/42): standard care (daily calcium, vitamin D, and regular exercise)	BMD (femoral neck, total hip, and lumbar spine [L1-4]). Biomarkers (CTX and OSC)	Femoral neck BMD: Significant positive change (+0.04%) was only observed in TCAG compared to the baseline, whereas CG group experienced a loss (−0.98%) (*p* = 0.05) Biomarker: significant positive change (−5.1%) in OSC was only observed in TCAG group (*p* = 0.03) compared to the baseline.
Woo et al. [41]	RCT	Hongkong, China (English)	120 community-living elderly people, aged 65 to 74	TC (60/58): Three sessions weekly for 12 months CG (60/59): original lifestyle	BMD (the total hip and spine [L1-4])	For female participants, TC vs. CG: total hip (Sig) (0.07 vs. −2.25%), spine (NS) (0.10 vs. 0.98%) For female participants, TC vs. CG: total hip (NS) (−0.48 vs. −0.15%), and spine (1.35 vs. 0.54%)
Zhou [44]	RCT	Shangxi, China (Chinese)	48 postmenopausal women (55.94 ± 2.83 years)	TCPH (12/12), Fan dancing (12/12), and walking (12/12): 45 to 60 min, 5 to 7 times weekly for 10 months. CG (12/12): original lifestyle	BMD (Lumbar spine [L2-4]	Lumbar spine (Sig) for TC vs. CG: (3.4 vs. −1.83%) and for TCPH vs. CG (1.84 vs. −1.83%)
Zhou [45]	RCT	Shanxi, China (Chinese)	60 postmenopausal women, aged 55.9	TC (12/12), TCPH (12/12), rope jumping (12/12), Mulan boxing (12/12): Five-to-seven sessions (45 to 60 min) for 10 months (Yang Style). CG (12/12): original lifestyle	BMD (Lumbar spine [L2-4], distal radius and ulna of wrist)	Mean change (Sig) for TCPH vs. CG: lumbar spine (0.035 vs. −0.019), distal radius (0.031 vs. −0.017), and distal ulna (0.033 vs. −0.016). Mean change (Sig) for TC vs. CG: lumbar spine (0.019 vs. −0.019), distal radius (0.017 vs. −0.017), and distal ulna (0.016 vs. −0.016)
Zhou et al. [46]	RCT	Shan Xi, China, (Chinese)	64 postmenopausal women, with a mean age of 57.21 ± 3.41	TCPH (16/16): 45 to 60 min, 5 to 7 times weekly for 6 months. CS (16/16): calcium carbonate 750 mg + calcium, 300 mg + Vitamin D, 100 IU, 1 tablet, 2 times daily for 6 months. TCPH + CSG: same as above CG: original lifestyle	BMD (Lumbar spine [L2-4]	TCPH + CSG vs. CSG: lumbar spine (Sig) (2.037 vs. 0.378%) TCPH vs. CG: lumbar spine (NS) (1.361 vs. −0.874%)
Zhou et al. [25]	RCT	Guizhou, China (Chinese)	40 older adults with hyperlipidemia (60 ± 5.6 years)	TC (20/20): four 90-min sessions for 6 months. CG (20/20): original lifestyle	BMD (Distal radius, Lumbar spine [L2-4] and trochanter) Biomarker (BGP)	Mean change (Sig) for TC vs. CG: distal radius (0.08 vs. −0.04), lumbar spine (0.05 vs. −0.05), trochanter (0.22 vs. −0.01) Biomarker: mean change (Sig) for TC (5.44) vs. CG (−0.11)

Abbreviation: RCT = Randomized controlled trial; TC = Taichi Quan; CG = control group; TCS = Taichi calcium supplement; CG1 = control group I (Dance); CG2= Control group II (walking); TCAG = Taichi adherence group (participants completed 75% of 9-month TC training); TCRT = Taichi resistance training (plus Taichi push hand); TCSB = Taichi softball; TCPH = Taichi push hand; DXA = dual-energy X-ray absorptiometry; CS = calcium supplement group; DEX = dual-energy X-ray densitometer; BUA = broadband ultrasound attenuation; SOS = speed of sound; tBMD = trabecular bone mineral density; iBMD = integral bone mineral density; CTD = cortical tissue density; CTX = C-terminal telopeptide of type I collagen (bone resorption); OSC = osteocalcin (bone absorption); BGP = Bone Gla protein; ALP = Alkaline Phosphase; PYD = pyridinoline; PTH = parathyroid hormone; TRAP = tartrate-resistant acid phosphatase; N-telopeptides of type I collagen; BRI = bone remodeling index; BAP = bone-specific alkaline phosphatase; Sig=significant difference with appropriate; NS = no significant difference.

**Table 2:** Study quality assessment for eligible randomized controlled studies.

Author	RE	RM	EC	OAB	WDR	SSE	ADA	TCID	QTCI	Study Quality
Chen et al. [20]	Yes	No	Yes	No	Yes	No	No	Yes	Yes	Fair
Chan et al. [35]	Yes	No	Yes	No	Yes	Yes	Yes	Yes	No	Fair
Du et al. [27]	Yes	No	Yes	No	Yes	No	No	Yes	Yes	Fair
Hui et al. [36]	Yes	Yes	Yes	Yes	Yes	Yes	Yes	Yes	Yes	Good
Mao [42]	Yes	No	Yes	No	Yes	No	No	Yes	Yes	Fair
Peppone et al. [26]	Yes	Yes	Yes	No	Yes	No	Yes	Yes	Yes	Good
Shen et al. [21]	Yes	Yes	Yes	Yes	Yes	No	Yes	Yes	Yes	Good
Shen et al. [37]	Yes	Yes	Yes	No	Yes	No	Yes	Yes	Yes	Good
Shen et al. [38]	Yes	Yes	Yes	Yes	Yes	No	Yes	Yes	Yes	Good
Song et al. [43]	Yes	No	Yes	No	Yes	No	No	No	Yes	Fair
Song et al [24]	Yes	Yes	Yes	No	Yes	Yes	Yes	Yes	Yes	Good
Song et al. [22]	Yes	No	Yes	No	Yes	No	No	Yes	No	Fair
Sufinowicz et al. [23]	Yes	No	No	No	Yes	No	No	Yes	No	Poor
Wang et al. [39]	Yes	No	Yes	No	Yes	No	Yes	Yes	Yes	Fair
Wayne et al. [40]	Yes	Yes	Yes	Yes	Yes	Yes	Yes	Yes	Yes	Good
Woo et al. [41]	Yes	Yes	Yes	No	Yes	Yes	Yes	No	No	Fair
Zhou [44]	Yes	No	Yes	No	Yes	No	No	Yes	No	Fair
Zhou [45]	Yes	No	Yes	No	Yes	No	No	Yes	No	Fair
Zhou et al [46]	Yes	No	Yes	No	Yes	No	No	Yes	No	Fair
Zhou et al. [25]	Yes	No	Yes	No	Yes	No	No	Yes	Yes	Fair

Abbreviation: RE = Randomization employed; RM = Randomization methods; EC = eligibility criteria; OAB = Outcome assessors blinded; WDR = Withdraw and dropouts reported; SSE = Sample size estimated; ADA = Appropriate data analysis; TCID = Taichi intervention described; QTCI = Qualification of TC instructor. Note: yes, design and methodology feature adequately described; No, design and methodology feature inadequately described; NA, not applicable.
